# The relationship between sleep quality, inappropriate medication use and frailty among older adults in aged care homes in Malaysia

**DOI:** 10.1371/journal.pone.0224122

**Published:** 2019-10-17

**Authors:** Suresh Kumar, Pei Se Wong, Syed Shahzad Hasan, Therese Kairuz

**Affiliations:** 1 Department of Pharmacy Practice, International Medical University, Kuala Lumpur, Malaysia; 2 Department of Pharmacy, University of Huddersfield, Huddersfield, West Yorkshire, United Kingdom; 3 School of Biological Sciences and Pharmacy, University of Newcastle, Newcastle, New South Wales, Australia; IRCCS Istituto Delle Scienze Neurologiche di Bologna, ITALY

## Abstract

Poor sleep quality is prevalent among older adults and is compounded by frailty and polypharmacy. This descriptive, cross-sectional study examines the associations between sleep quality, inappropriate medication use and frailty. The study was conducted among 151 residents of 11 aged care homes in three states in Malaysia; convenience sampling was used. Subjective sleep quality was assessed using the Pittsburgh Sleep Quality Index (PSQI), and Groningen Frailty Indicator (GFI) was used to assess frailty. Medication appropriateness was assessed using Drug burden Index (DBI), Potentially Inappropriate Medications (PIMs) and Potentially Inappropriate Prescriptions (PIPs). Most of the subjects (approximately 95%) reported poor sleep quality, as measured by a cut-off of global PSQI score of ≥ 5. With a second cut-off at 10, just over half (56%) reported moderately poor sleep quality followed by 39% who had very poor sleep quality. Most (90%) denied taking medication to improve their sleep during the previous month. There was no statistically significant association between medication inappropriateness (PIMs, PIPs, DBI) and global PSQI score. However, the average number of PIM was associated significantly with sleep efficiency (a measure of the actual ‘sleep to total time spent in bed) (p = 0.037). The average number of PIP was associated with subjective sleep quality (p = 0.045) and the use of sleep medications (p = 0.001), and inversely associated with sleep disturbance (0.049). Furthermore, frailty correlated significantly with poor overall sleep quality (p = 0.032). Findings support the need for medication review to identify and reduce PIMs and optimise prescriptions to improve sleep quality and hence, related health outcomes among residents of aged care homes.

## Introduction

Ageing is an inevitable process which is associated with many health issues. Worldwide, the number of older people (aged ≥60) is expected to double by 2050 and more than triple by 2100, while the number of persons ≥80 years is projected to increase almost 7 times [1]. The trend is reflected in Asia where people aged ≥60 years as expected to increase from 508 million in 2015 (or 11.6% of the total population) to 1,294 million by 2050 (or 24.6% of the total population) [1].

In Malaysia, according to the revised population projection, the proportion of the aged population is expected to increase from 5% (2010) to 14.5% in 2040, with the dependency ratio increasing threefold to 21.7% in 2040 (21.7%) [2].

Chronic and complex multi-morbidities accompany the ageing process, and older people generally require more drugs to treat comorbid conditions. Multiple medical conditions and polypharmacy may increase the risk of adverse drug reactions in older people. Prescribing for older people is a complex process that may lead to the prescribing of Potentially Inappropriate Medications (PIMs) or give rise to Potentially Inappropriate Prescriptions (PIPs) [3]. The Beer Criteria for PIMs [4] and STOPP criteria for PIPs [5] refer to the guidelines for healthcare professionals to help improve the safety of prescribing medications for older adults and are based on a medicine-to-avoid list compiled by expert consensus.

Various instruments are available to identify PIMs, such as (updated) Beers’ criteria [4] and the Screening Tool of Older Person’s Prescriptions (STOPP) [5,6]. Beer’s criteria include drugs considered as inappropriate for use in the elderly, such as oral decongestants, stimulants and theobromines, and strongly recommend avoiding the use of benzodiazepines, non-benzodiazepines with affinity for benzodiazepine receptors and anticholinergics, as well as H2 receptor antagonists, anticonvulsants and antipsychotics. Benzodiazepines and anticholinergics contribute to medication burden. Older people with dementia or cognitive impairment, or with history of falls or fractures, are particularly vulnerable to the effects of these drugs [5]. Unfortunately, benzodiazepines are the most frequently prescribed medications for the management of sleep disorders in older people; this is despite their potential risk for adverse effects and ineffectiveness in maintaining sleep quality, and their [7–9]. Medications used to manage chronic medical conditions may contribute to sleep disturbances, such as antidepressants, antihypertensives, and drugs to treat parkinsonism [5,10]. In addition, older people tend to self-medicate with over-the-counter (OTC) product which often have sedative or anticholinergic effects [11].

Ageing is associated with difficulty in falling asleep, maintaining sleep, sleep fragmentation, early waking hours, and day time sleeping. Poor sleep quality includes variations in sleep duration and sleep pattern, and disturbed sleep is associated with poor health outcomes. Consequences of poor sleep quality include cognitive impairment, poor social functioning, accidents, increased risk of falls, and daytime fatigue, as well as a decline in physical and mental health, and health-related quality of life. Sleep disorders are associated with increased health care visits, and a high economic burden on individuals as well as society [10–12].

Polypharmacy and inappropriate medications may affect sleep and reduce health-related quality of life although there is a lack of evidence on how medication burden and PIMs are associated with sleep quality. Therefore, the aims of this study were to determine sleep quality among older people in residential aged care homes, and to examine associations of sleep quality with inappropriate medication use and frailty.

## Methods

### Ethical approval

The study was approved by the Joint Research and Ethics Committee at the International University of Malaysia [Research ID: IMU 385/2017], and the management of participating aged care homes. Results are de-identified and no personal data are reported.

### Sampling frame

This cross-sectional study assessed sleep quality, medication appropriateness, and physical functioning among older residents, between July and September 2017, from 11 aged-care facilities in suburban peninsular Malaysia (from three states Kuala Lumpur, Selangor and Perak). Based on an estimation of the population aged ≥60 years (6%, 2016), the required sample size was 135 residents [3,13,14]. Inclusion criteria were: ≥ 60 years of age, mobile, having at least 1 long-term medical condition, receiving at least 1 long-term medication, able to articulate and provide verbal or written consent to participate.

### Sampling procedure

Consenting residential aged care homes in suburban peninsular Malaysia were selected using convenience sampling. Information was provided to residents prior to obtaining their written consent. An interviewer-administered Comprehensive Assessment Form (CAF) contained questionnaires which were used to assess sleep quality, medication inappropriateness and frailty. Sociodemographic characteristics included age, gender, ethnicity, education, occupation, marital status and medical history.

### Assessment of sleep quality

Subjective sleep quality was measured using the Pittsburgh Sleep Quality Index (PSQI) which differentiate between good and poor sleepers [15–19]. The PSQI tool measures 7 components of sleep quality: subjective sleep quality, sleep latency, sleep duration, habitual sleep efficiency, sleep disturbances, use of sleeping medications, and daytime dysfunction. The PSQI instrument consists of 19 self-rated questions and 5 questions rated by a roommate (where applicable). The 19 items are grouped into 7 domains, each with scores of 0–3, and weighted equally. The Global PSQI score is calculated by adding the individual component scores and ranges from 0–21; the higher the score, the poorer the quality of sleep. Subjects with global PSQI scores >5 are considered to have poor sleep quality.

The psychometric properties of the PSQI tool are reliable with internal consistency reliability ranging from 0.80 to 0.88, test-retest reliability from 0.85 to 0.87 and good sensitivity and specificity in those with or without sleep problems when estimated at a cut-off score of 5 [15–19]. To obtain more insight into individuals’ sleep quality, we introduced a second cut-off score of 10; this identified those who have very poor sleep quality. In summary, subjects with PSQI scores ≤5 have normal sleep quality (NSQ), scores 5–10 indicate moderately poor sleep quality (MPSQ) and scores 10 to 21 indicate very poor sleep quality (VPSQ).[20]

### Measurement of frailty

Frailty was evaluated using the Groningen Frailty Indicator (GFI). The GFI, validated in many institutional homes, comprises fifteen dichotomous items with a score range from 0 (normal activity without restriction) to 15 (completely disabled). GFI assesses individual’s loss of functions and resources in 4 domains: physical (mobility functions, multiple health problems, physical fatigue, vision, hearing), cognitive (cognitive dysfunction), social (emotional isolation), and psychological (depressed mood and feelings of anxiety). Subjects with a score of ≥4 are considered frail [21,22]. The GFI construct was found to be valid and had good internal consistency in assessing frailty among aged care home residents (Cronbach’s alpha = 0.77) [23].

### Medication and appropriateness and burden

Medication data were gathered from the participants’ medical records. Medication appropriateness was assessed using Beers criteria (2015) and STOPP criteria (2015) [5,6,24] to identify PIMs and PIPs. For the purposes of this study polypharmacy is defined as taking ≥5 concurrent medications [25].

‘Medication burden’ refers to the use of sedatives and medications with anticholinergic properties. The validated Drug Burden Index [DBI = D/ (D+ δ)] was used to estimate drug burden, where D is the daily dose taken by the individual and δ is the minimum efficacious dose [26]; doses were those approved by, and registered with, the Ministry of Health Malaysia (Formulari Ubat KKM) [27]. The dosing instructions were obtained from available medical records. The Malaysian product information and Monthly Index of Medical Specialities [28] (MIMS Malaysia, paperback 2017) were used to identify medications with clinically significant anticholinergic and/or sedative effects. DBI calculations did not include complementary medications, health supplements and medications prescribed on a PRN basis.

### Statistical analysis

Statistical analysis was performed using SPSS (version 24) with a significance level of 0.05. The data are presented as percentages or means and standard deviations. Chi-square test was used to examine independence between variables (e.g. polypharmacy and sleep quality; frailty and sleep quality). Spearman’s correlation was used to determine underlying relationships between sleep quality, drug burden and frailty.

## Results

### Socio-demographic characteristics

A total of 151 older adults residing in aged care homes were interviewed. The mean age of the study population was 74.5 ± 8.4 years, with Chinese (98%) as the major ethnic group. The sample population comprised 77 (51%) males and 74 (49%) females. The majority of study participants were either unmarried or not living with their partner. The study population had a relatively little education; most subjects had primary school education or less. Socio-demographic characteristics of the study population are included in “Table 1”.

**Table 1:** Demographic characteristics of study participants by sleep quality status.

Variable	Value	Range	Possible range
Mean age (SD)	74.5 (8.4)	60–98	-
No of male participants (%)	77 (51.0)	**-**	**-**
No of Chinese participants (%)	148 (98.0)	-	-
No of married participants (%)	50 (37.6)	-	-
Participants with > primary schooling (%)	67 (44.4)	-	-
Physical activity, moderate level	35 (23.2)	-	-
**Mean PSQI-Latency in min (SD)**	64.3 (39.9)	5.0–183.0	-
**Mean PSQI-efficiency (SD)**	68.6 (20.5)	15.5–100	0–100
**Mean PSQI-duration in min (SD)**	346.8 (90.6)	120.0–600.0	-
**Mean PSQI-disturbances (SD)**	6.3 (2.1)	0.0–12.0	0–27
**Mean PSQI-sleep quality (SD)**	1.1 (0.7)	0.0–3.0	0.0–3.0
Very good, n (%)	23 (15.1)	-	-
Fairly good, n (%)	97 (63.8)	-	-
Fairly bad, n (%)	25 (16.4)	-	-
Very bad, n (%)	7 (4.6)	-	-
**Mean PSQI-daytime dysfunction (SD)**	2.9 (1.6)	0.0–6.0	0.0–6.0
**PSQI-sleep medication**			
Not during the past month, n (%)	136 (89.5)	-	-
Less than once a week, n (%)	12 (7.9)	-	-
Once or twice a week, n (%)	1 (0.7)	-	-
Three or more times a week, n (%)	3 (2.0)	-	-
**Mean PSQI-Total (SD)**	9.7 (2.7)	4.0–15.0	0–21
**PSQI Classification 1**			
Good sleep (0–5), n (%)	7 (4.6)	-	-
Poor sleep (>5), n (%)	145 (95.4)	-	-
**PSQI Classification 2**			
Normal Sleep Quality (NSQ), n (%)	7 (4.6)	0–5	
Moderately Poor Sleep Quality (MPSQ), n (%)	86 (56.6)	5–10	
Very Poor Sleep Quality (VPSQ), n (%)	59 (38.8)	10–21	

PSQI: Pittsburgh Sleep Quality Index

### Sleep quality of study participants

Most (95%) of the sample population reported poor sleep quality as measured by global PSQI score cut-off of 5 (Table 1). By adopting the second cut-off at 10, only 5% of the individuals had normal sleep quality, just over half (56%) had moderately poor sleep quality and more than one third (39%) had very poor sleep quality. Average sleep efficiency (percentage of time spent asleep while in bed) was 69% (±20%) with a range of 15%– 100%. The sleep latency (amount of time it takes to fall asleep) ranged from 5 to 183 minutes (average 64 minutes). Sleep duration (total amount of time spent asleep) ranged between 2 to 10 hours (average 5 hours and 46 minutes). The average sleep disturbance score (6.32) ranged between 0 to 12 (possible range: 0 to 27) where 0 indicate the absence of sleep disturbances and 27 indicates inadequate sleep due to severe sleep disturbances.

More than three quarters of this population (79%) rated subjective sleep quality either as very good or fairly good; the remainder rated it either fairly poor or very poor. Most subjects (90%) denied taking medication to improve sleep during the previous month. Associations between participants’ health outcomes and sleep quality are included in “Table 2”.

**Table 2:** Medication-related factors and frailty by sleep quality.

Variables	Total value	NSQ Value n = 7	MPSQ Value n = 86	VPSQ Value n = 58	p-value
No. of medications: mean (SD)	3.5 (1.9)	2.6 (1.7)	3.6 (2.0)	3.6 (1.9)	0.181
Polypharmacy: n (%)	41 (27.1)	1 (14.3)	23 (26.7)	17 (29.3)	0.433
Exposed to a PIM (n, %)	49 (32.2)	2 (28.6)	26 (30.2)	21 (36.2)	0.822
No. of PIM: mean (SD)	0.4 (0.7)	0.12 (0.2)	0.11 (0.2)	0.2 (0.3)	0.790
Exposed to a PIP (n, %)	52 (34.2)	1 (14.3)	28 (32.6)	23 (39.7)	0.251
Total No. of PIP: mean (SD)	0.5 (0.8)	0.17 (0.2)	0.16 (0.4)	0.3 (0.1)	0.945
DBI > 0 (n, %)	112 (73.7)	6 (85.7)	61 (70.9)	45 (77.6)	0.459
DBI: mean (SD)	0.8 (0.6)	1.1 (0.6)	0.7 (0.6)	0.9 (0.6)	0.280
GFI Score, mean (SD)	-	2.3 (2.4)	4.48 (2.8)	4.9 (2.5)	**0.023**
Frail (score ≥ 4), n (%)	-	4 (57.1)	65 (75.6)	45 (77.6)	0.533

### Medication appropriateness and sleep quality

Just over one quarter (27%) of the study population exhibited polypharmacy (≥ 5 medications); participants were taking an average of 3.5 medications per person. Nearly one third (29%) with very poor sleep quality (VPSQ) were taking ≥ 5 medications compared with just over one quarter (27%) who had moderately poor sleep quality (MPSQ) and 14% who reported normal sleep quality (NSQ) (Table 2).

Almost one third (32%) were exposed to PIMs, with an average PIM (SD) of 0.4 (0.7) per participant. A larger proportion (36%) of those with VPSQ were exposed to PIMs compared to 30% for MPSQ and 28% for NSQ. Similarly, about 40% of the participants with VPSQ were exposed to PIPs compared to 33% for MPSQ and 14% for NSQ. Overall, nearly three quarter (74%) were exposed to medications with anticholinergic or sedative effects with a mean DBI (SD) of 0.79 (0.63). However, no significant differences in DBI were observed between NSQ, MPSQ and VPSQ groups.

We observed a significant correlation between medication appropriateness and some sleep quality components. There was a positive and statistically significant correlation between sleep efficiency and the average number of PIMs (+0.170, *p* = 0.037), and the average number of PIPs was found to be significantly correlated with subjective sleep quality (+0.162, p = 0.047) and sleep medication use (+0.263, p = 0.001); however, PIPs was negatively correlated with sleep disturbance (-0.161, p = 0.049) in this population.

There was a positive and statistically significant correlation (+0.175, *p* = 0.032) between global PSQI score and frailty. However, the present study did not observe significant relationships of medication appropriateness with other health outcomes (*p* value>0.05). Correlation coefficients between sleep quality and medication-related parameters are reported in “Table 3”.

**Table 3:** Relationships between Global PSQI score, medication-related variables and frailty.

Items	Global PSQI Score		
	N	Coefficient	p-value
**Medication-related variables**			
No. of medications	151	0.103	0.207
No. of PIM (Beer’s Criteria)	150	0.150	0.067
No. of PIP (STOPP criteria)	150	0.045	0.583
Drug Burden Index	151	0.098	0.232
**Frailty**			
GFI Score	151	0.175	**0.032**

### Sleep quality and frailty

Most participants (75.5%) were categorised as frail (GFI ≥ 4). The study population with very poor sleep quality (VPSQ) had the highest mean GFI score (4.9 ± 2.5), followed by participants with moderately poor sleep quality (MPSQ) (4.5 ± 2.8) and participants with normal sleep quality (NSQ) (2.3 ± 2.4). These differences in GFI scores were statistically significant (p = 0.023).

## Discussion

This study examined the associations between medication inappropriateness, including exposure to sedative medications and those with anticholinergic properties, and components of sleep quality. The study also attempted to establish a relationship between sleep quality and frailty, a physical health outcome.

Sleep quality exposed can be measured subjectively, objectively or by a combination of both methods. We used a subjective measurement (PSQI) as PSQI global scores are known to correlate well with sleep diaries in older adults [20]. The global sleep quality score for most (95%) of the study population was >5, which indicates poor sleep quality. Findings are consistent with a study conducted in Malaysia by Azri et al. (2016) which reported that more than 70% of institutionalised adults had poor sleep quality [29]. Prevalence of poor sleep quality is high when compared to studies conducted in other countries such as Iran [30] and Turkey [31]. The high proportion of participants with poorer sleep quality in our study may be due to the presence of chronic diseases, and social and environmental factors as people residing in (elderly) care homes often have poor social relationships; institutionalisation may reflect a lack of family support. Environmental factors such as noise, light, temperature and interruptions by staff may also affect sleep quality in institutions [11].

The current study established a relationship between GFI scores and sleep quality. It was found that the majority of participants had a GFI score of ≥4, averaging 4.69 for poor sleepers and 2.27 for ‘good’ sleepers. Physical inactivity is a contributing factor for poor sleep quality [32], and there is also evidence to suggest an association with quality of life in the elderly [31,33–35]. Older people in aged care homes who have low quality of life would be expected to have poor sleep quality if they experienced a lack of psycho-social support.

A study conducted in Malaysian care homes in 2017 reported a frailty score of 6.4 [35], indicating that residents were more frail than residents in the current study. Our data suggest that increased frailty is associated with poorer sleep quality among elderly people residing in care homes. Even though evidence correlates sleep quality with physical activity, quality of life, and chronic disease, to the best of our knowledge the current study is the first to correlate sleep quality with frailty in older people residing in aged care homes.

### Medications and drug burden

Sedative medications and medicines associated with anticholinergic effects are commonly prescribed to manage poor sleep, and behavioural and psychological symptoms associated with dementia; this occurs despite their association with falls and stroke [8,36–39]. The RedUse trial conducted in Australian residential aged care homes found that nearly half (41%) of the study population were prescribed antidepressants, almost one third (30%) were taking benzodiazepines, and 10% received antipsychotics, indicating a large drug burden among the population [8]. We analysed the drug burden in our study and calculated the DBI, correlating it with sleep quality. DBI provides insight into the summative effects of anticholinergic and sedative effects in the elderly. The mean DBI in the study population was 0.79 which was comparable to similar studies conducted in Australian residential aged care homes [40,41].

There is an association between DBI and hospital admissions, frailty, falls, poorer physical functioning and death; the index increases with the number of hospital admissions, increased frailty, more falls, and poorer physical functioning. Although studies associated the health outcomes and physical function of aged care residents, there is a lack of evidence on how DBI is associated with sleep quality. There was no significant association between DBI and sleep quality I the current study.

For the purpose of this study, polypharmacy was considered as concurrent use of more than five medications (including prescription and non-prescription medicines). Participants with MPSQ and VPSQ were exposed to a higher average number of medications (>3.5) when compared to NSQ (2.57). However, we could not find any statistically significant relationship between polypharmacy and sleep quality, and further investigations are recommended, particularly how DBI and polypharmacy affect subjective sleep quality over a long period of time.

### Inappropriate medication use

There is evidence that sleep quality correlates with chronic disease [33], depression [42,43] and sleep medications [9,39,44,45]. The majority of our study population who were exposed to PIMs were taking antipsychotics or antihistamines with sedative or anticholinergic side effects. These medications are associated with cognitive impairment, sleep disturbance including excessive sleepiness, and increased risk of falls and fractures [46,47]. To the best of our knowledge, the current study is the first to correlate sleep quality with inappropriate medication use. However, we could not find any association between global sleep quality and average number PIMs (Beer’s criteria) or PIPs (STOPP Criteria). Although the average PIM and PIP values were not associated (statistically) with the global sleep quality score they were associated with some components of sleep quality: PIMs were associated with subjective sleep efficiency, a measure of the actual ‘sleep to total time spent in bed’. Participants taking more PIMs tended to have less sleep efficiency. PIPs were associated with subjective sleep quality and the use of sleep medications, and inversely associated with sleep disturbance. The use of medications with sedative and anticholinergic effects may contribute to this inverse relationship as they could cause sedation and excessive daytime sleepiness. These effects may lead to poor sleep quality, particularly in the frail elderly; however, further research may be required to establish an explanation for the inverse relationship.

Participants with a greater number of inappropriate prescriptions reported poor subjective sleep quality and they tended to use prescription or over- the-counter (OTC) sleep medications. PIMs could be contributing to sleep problems in this population necessitating the use of additional hypnotics or OTC medications; this may lead to increased exposure to PIMs and adverse effects. Rational prescribing that weighs potential risk against perceived benefits is highly recommended to improve sleep quality, and reduce cognitive impairments, risk of falls and hospital admissions, thereby improving the overall quality of life of older people in residential care. Findings suggest a need for medication reviews to avoid medication inappropriateness among older people residing in aged care homes.

### Limitations

There were limitations in this study that need to be considered when interpreting the results, including convenience sampling (i.e. non probability sampling) which may lead to selection bias. There may have been recall bias associated with the use of the PSQI instrument. Although it provides comprehensive information about subjective sleep quality, the use of objective measurements, such as technology-enabled actigraphy, may provide deeper understanding about the quality of sleep.

Malaysia is a multi-ethnic country, with three major races, Malay, Chinese and Indians. Consent for including residential facilities was provided by the Management of the respective aged care homes; most of these homes had Chinese residents and the small number of non-Chinese participants limits generalizability to the (whole) Malaysian population.

## Conclusions

Sleep is necessary maintain health and quality of life among older people, and reduced quality of sleep may result in poor health outcomes. Most residents in this study reported poor sleep quality, and PIMs and PIPs affected certain components of sleep quality. Increased frailty may be a physical health-related factor contributing to poorer sleep quality. Older people are exposed to multiple medications to manage comorbid conditions and longitudinal studies are recommended to determine how physical health parameters, PIMs and medication burden affect sleep quality of older people.

## Supporting information

S1 Minimal anonymized data set (XLSX)
